# Hepatocyte-Specific *Arid1a* Deficiency Initiates Mouse Steatohepatitis and Hepatocellular Carcinoma

**DOI:** 10.1371/journal.pone.0143042

**Published:** 2015-11-16

**Authors:** Jia-Zhu Fang, Chong Li, Xiao-Yan Liu, Tao-Tao Hu, Zu-Sen Fan, Ze-Guang Han

**Affiliations:** 1 Key Laboratory of Systems Biomedicine (Ministry of Education) and Collaborative Innovation Center of Systems Biomedicine of Rui-Jin Hospital, Shanghai Jiao Tong University School of Medicine, Shanghai, China; 2 Shanghai-MOST Key Laboratory for Disease and Health Genomics, Chinese National Human Genome Center at Shanghai, Shanghai, China; 3 Shanghai Center for Systems Biomedicine, Shanghai Jiao Tong University, Shanghai, China; 4 Chinese Academy of Sciences Key Laboratory of Infection and Immunity, Institute of Biophysics, Chinese Academy of Sciences, Beijing, China; University of Medicine, Greifswald, GERMANY

## Abstract

*ARID1A*, encoding a subunit of chromatin remodeling SWI/SNF complexes, has recently been considered as a new type of tumor suppressor gene for its somatic mutations frequently found in various human tumors, including hepatocellular carcinoma (HCC). However, the role and mechanism of inactivated *ARID1A* mutations in tumorigenesis remain unclear. To investigate the role of *ARID1A* inactivation in HCC pathogenesis, we generated hepatocyte-specific *Arid1a* knockout (*Arid1a* ^*LKO*^) mice by crossing mice carrying loxP-flanked *Arid1a* exon 8 alleles (*Arid1a* ^*f/f*^) with albumin promoter-Cre transgenic mice. Significantly, the hepatocyte-specific *Arid1a* deficiency results in mouse steatohepatitis and HCC development. In *Arid1a* ^*LKO*^ mice, we found that innate immune cells, including F4/80+ macrophages and CD11c+ neutrophil cells, infiltrate into the liver parenchyma, accompanied by the increased tumor necrosis factor (TNF)-α and interleukin (IL)-6, and activation of STAT3 and NF-κB pathways. In conclusion, hepatocyte-specific *Arid1a* deficiency could lead to mouse steatohepatitis and HCC development. This study provides an alternative mechanism by which *Arid1a* deficiency contributes to HCC tumorigenesis.

## Introduction

*ARID1A*, also known as BAF250a, is a subunit of the SWI/SNF complex. *ARID1A* mutations, including missense, nonsense and frame shift mutations led by small insertion and deletion, have frequently been detected in a series of human tumors, including ovarian clear-cell carcinoma [1, 2], gastric cancer [3, 4], breast cancer [3, 5, 6], pancreatic cancer [3, 7, 8], cholangiocarcinoma [9, 10], clear cell renal cell carcinoma [11], esophageal adenocarcinoma [12], neuroblastoma [13], diffuse large B-cell lymphoma [14] and transitional cell carcinoma of the bladder [15]. Patients with *ARID1A* mutations may constitute a specific subtype of certain tumors. For example, *ARID1A* mutations were frequently identified in gastric cancers with microsatellite instability and Epstein-Barr virus infection [16]. In addition to somatic mutations, *ARID1A* loss has also been found in a variety of human tumor types, such as uterine endometrioid carcinomas, uterine clear-cell carcinomas, uterine serous carcinomas, uterine carcinosarcomas, clear cell renal cell carcinoma, prostate cancers and medulloblastomas [3]. Recently, several groups, including ours, detected *ARID1A* mutations in 10–15% of hepatocellular carcinoma (HCC) [17–20]. *ARID1A* has recently been suggested to be a new type of tumor suppressor gene in many tumors; however, the role of and mechanism underlying *ARID1A* mutation or loss in HCC tumorigenesis remain unclear.

Previous *in vitro* experiments supported the idea that *ARID1A*/BAF250a exerts a tumor suppressive effect. *ARID1A* knockdown promotes cell cycle progression, cell proliferation, tumorigenicity, migration, invasion and metastasis [4, 21–23], whereas *ARID1A* overexpression inhibits cell proliferation and tumor growth [4, 21]. *In vivo* study also shows that *ARID1A* deficiency could promote tumor formation in ovary cancer [24, 25]. However, *in vivo* evidence that ARID1A functions as a tumor suppressor in HCC has not yet been provided. Previous studies using genetically engineered *Arid1a*-deficient mice demonstrated that *Arid1a* is required for animal development. Deletion of *Arid1a* leads to developmental arrest and the absence of the mesodermal layer [26]. Conditional *Arid1a* ablation in mice hearts results in trabeculation defects and embryonic lethality [27]. Here, we established hepatocyte-specific *Arid1a*-deficient mice and found that these mice spontaneously developed steatohepatitis and HCC. To our knowledge, we have developed the first murine *Arid1a* deficiency-induced HCC model.

## Materials and Methods

### Mice

LoxP-flanked (floxed [f]) *Arid1a* (*Arid1a* ^*f/f*^) mice were kindly provided by Zhong Wang at the Cardiovascular Research Center, Massachusetts General Hospital, Harvard Medical School. *Arid1a* ^*f/f*^ mice and albumin promoter (Alb)-Cre (from the Jackson Laboratory) were crossed to generate conditional tissue-specific *Arid1a*-KO mice designated as *Arid1a* ^*LKO*^. In all of the animal experiments, *Arid1a* ^*f/f*^ littermates lacking Cre recombinase were used as controls. All animals received care according to the ethical guidelines of Shanghai Bimodel Organism Science & Technology Development Co. Ltd., and all animal procedures were conducted in compliance with institutional guidelines and protocols. This project was approved by the ethics committee of the Chinese National Human Genome Center at Shanghai (IACUC no. 2012–0026).

### Genotyping

Mice genotyping were described as before [26]. In brief, mice liver DNA or HCC DNA-harvested by LCM (Laser capture microdissection) were prepared with Tiagen Tissue Genome DNA Extraction Kit (Tiagen, Beijing, China). DNA was diluted to 100 ng/μl. 1μl DNA was employed as the templates in a 20 μl system. Primers were listed in Table 1. The PCR products were electrophoresis in 1% agarose gel for 20 min, and recorded by Gel imaging analysis system. For *Arid1a* gene, the 812-bp band indicates *Arid1a* ^*f/f*^ while the 268-bp band indicates *Arid1a* ^*LKO*^. The PCR product of *Cre* is the 300-bp band.

**Table 1:** Primers for genotyping.

Genes	Sequence (5'-3')
*Arid1a-F* ^#^	GTAATGGGAAAGCGACTACTGGAG
*Arid1a-R* ^$^	TGTTCATTTTTGTGGCGGGAG
*cre-F*	GTAATGGGAAAGCGACTACTGGAG
*cre-R*	TGTTCATTTTTGTGGCGGGAG

^#^F, Forward;

^$^R, Reverse.

### Diethyl nitrosamine (DEN)-induced hepatocarcinogenesis murine model

Two week-old male *Arid1a* ^*f/f*^ and *Arid1a* ^*LKO*^ mice were injected with DEN (25 mg/kg of body weight) intraperitoneally (*i*.*p*.). Mice were subsequently sacrificed at 4 or 9 months and tumor nodules on the liver surface were calculated. The livers of these mice were also routinely formalin-fixed and paraffin-embedded for further analysis. The body and liver weights were analyzed and their peripheral blood sera were harvested.

### LPS induced acute liver injury

Acute liver injury was induced as described before [28]. In brief, after injection of LPS (25 μg/kg; From Escherichia coli 0111:B4, Sigma-Aldrich, Germany) *i*.*p*., the mice body temperature (BT) were observed carefully by infrared thermometer at 12h, 24h, 48h and 72h. The BT < 23.4°C as humane endpoint was implemented [29]. The bloods were collected for aminotransferases (ALT) analysis and right liver lobe was fixed for pathologic analysis. Remaining parts of liver were stored at -80°C.

### Histochemistry and immunohistochemical (IHC) staining

Hematoxylin and eosin (H&E) and Sirius red collagen staining were performed using a standard protocol for paraffin sections. Cryosections were used for Oil red O staining according to a standard protocol. IHC staining was performed on paraffin sections using a rabbit polyclonal antibody that had been raised against Ki-67, PCNA, F4/80 (Cell Signaling Technology). Primary antibodies were incubated at 4°C overnight. The staining was visualized using EnVision^™^ Detection Systems (Dako Corp.).

### Laser capture microdissection

Tumor tissues were embedded in OCT (Sakura, Hayward CA). Three 10 μm-thick cryosections were prepared and air dried. PixCell Iie system (Arcturus Engineering, Mountain View CA) were employed to perform LCM according to the manufactures protocol [30, 31].

### Scoring system definition for pathological examination

The steatohepatitis was evaluated by a standard criteria scoring system as described before [32, 33]. In brief, steatosis area percent: 0 (0–5%), 1 (5%-33%), 2 (33%-66%) and 3 (> 66%). Ballooning degeneration distribution: 0 (absence), 1 (scattered) and 2 (panacinar). Lobular inflammation: 0 (absence), 1 (1–2 foci), 2 (2–4 foci) and 3 (>4 foci) at 200 field. Portal inflammation was graded as 0 (none), 1 (mild or few), 2 (moderate) and 3 (marked or many). All sections were evaluated blindly by two pathologists.

### Nonparenchymal liver cells (NPC)

NPCs were prepared as previously described [34]. In brief, the livers were perfused with 1 × Hanks’, then passed through a 80 μm mesh in RPMI 1640 medium (2% FBS, Invitrogen). Collected liver cell suspensions were centrifuged (2 minutes, 48g). Transfer the supernatant to a fresh tube and centrifuge (10 minutes, 440g). The pellet were resuspended with 40% Percoll^®^, and mounted on 80% Percoll^®^. NPCs were at the interface after gradient centrifuge (15 minutes, 780g).

### Flow cytometric analysis

FACSCalibur flow cytometer (BD Biosciences) was employed for flow cytometry. Firstly, NPCs’ Fcγ III/II receptor were blocked by anti-CD16/CD32 antibodies. Then, incubated with anti-CD11c, anti-CD3, anti-CD19 and anti-F4/80 antibodies (eBiosciences), respectively. The data was analyzed with CELLQuest software (BD Biosciences).

### Quantitative real-time RT-PCR

Total RNAs of mouse livers were extracted using TRIzol^®^ (Invitrogen). cDNA was reverse transcripted with the SuperScript First-Strand Synthesis System (Invitrogen). Real-time PCR was performed in 20μl volume containing SYBR Green Reagent (TaKaRa) and specific primers (Table 2) on a qPCR machine (TaKaRa). The reactions were in triplicate. All of the results were normalized to GAPDH mRNA levels.

**Table 2:** Primers for cytokines and chemokines.

Genes	Sequence (5'-3')
*mIL-6-F*	AGATAACAAGAAAGACAAAGCCAGAGTC
*mIL-6-R*	GCATTGGAAATTGGGGTAGGAAG
*mTNF-F*	GAGTGACAAGCCTGTAGCCC
*mTNF-R*	GGAGGTTGACTTTCTCCTGGTAT
*mIFN-γ-F*	ACACTGCATCTTGGCTTTGCAGCT
*mIFN-γ-R*	TGAGCTCATTGAATGCTTGGCGCT
*mCCL1-F*	GGCTGCCGTGTGGATACAG
*mCCL1-R*	AGGTGATTTTGAACCCACGTTT
*mCCL11-F*	GAATCACCAACAACAGATGCAC
*mCCL11-R*	ATCCTGGACCCACTTCTTCTT
*mCCL12-F*	ATTTCCACACTTCTATGCCTCCT
*mCCL12-R*	ATCCAGTATGGTCCTGAAGATCA
*mCCL17-F*	TACCATGAGGTCACTTCAGATGC
*mCCL17-R*	GCACTCTCGGCCTACATTGG
*mCCL19-F*	GGGGTGCTAATGATGCGGAA
*mCCL19-R*	CCTTAGTGTGGTGAACACAACA
*mCCL2-F*	ATTCTGTGACCATCCCCTCAT
*mCCL2-R*	TGTATGTGCCTCTGAACCCAC
*mCCL20-F*	GCCTCTCGTACATACAGACGC
*mCCL20-R*	CCAGTTCTGCTTTGGATCAGC
*mCCL4-F*	TTCCTGCTGTTTCTCTTACACCT
*mCCL4-R*	CTGTCTGCCTCTTTTGGTCAG
*mCCL5-F*	GCTGCTTTGCCTACCTCTCC
*mCCL5-R*	TCGAGTGACAAACACGACTGC
*mCCL6-F*	GCTGGCCTCATACAAGAAATGG
*mCCL6-R*	GCTTAGGCACCTCTGAACTCTC
*mCCL7-F*	GCTGCTTTCAGCATCCAAGTG
*mCCL7-R*	CCAGGGACACCGACTACTG
*mCCL8-F*	TCTACGCAGTGCTTCTTTGCC
*mCCL8-R*	AAGGGGGATCTTCAGCTTTAGTA
*mCCL9-F*	CCCTCTCCTTCCTCATTCTTACA
*mCCL9-R*	AGTCTTGAAAGCCCATGTGAAA
*mCXCL11-F*	GGCTTCCTTATGTTCAAACAGGG
*mCXCL11-R*	GCCGTTACTCGGGTAAATTACA
*mCXCL13-F*	GGCCACGGTATTCTGGAAGC
*mCXCL13-R*	GGGCGTAACTTGAATCCGATCTA
*mCXCL10-F*	CCAAGTGCTGCCGTCATTTTC
*mCXCL10-R*	GGCTCGCAGGGATGATTTCAA
*mCXCL12-F*	TGCATCAGTGACGGTAAACCA
*mCXCL12-R*	TTCTTCAGCCGTGCAACAATC
*mCXCL2-F*	CCAACCACCAGGCTACAGG
*mCXCL2-R*	GCGTCACACTCAAGCTCTG
*mCXCL5-F*	TGCCCTACGGTGGAAGTCATA
*mCXCL5-R*	TGCATTCCGCTTAGCTTTCTTT
*mCXCL9-F*	GGAGTTCGAGGAACCCTAGTG
*mCXCL9-R*	GGGATTTGTAGTGGATCGTGC
*mPROM1-F*	CCTTGTGGTTCTTACGTTTGTTG
*mPROM1-R*	CGTTGACGACATTCTCAAGCTG
*mDLK1-F*	CCCAGGTGAGCTTCGAGTG
*mDLK1-R*	GGAGAGGGGTACTCTTGTTGAG
*mAFP-F*	CTTCCCTCATCCTCCTGCTAC
*mAFP-R*	ACAAACTGGGTAAAGGTGATGG
*mGAPDH-F*	TGTGTCCGTCGTGGATCTGA
*mGAPDH-R*	CCTGCTTCACCACCTTCTTGA

### Liver function test

ALT, AST, TCHO, HDL-C and LDL-C were measured using standard procedures, as previously described [35].

### Immunoblot analyses

Protein lysates from mouse livers were separated using SDS-PAGE, and transferred to nitrocellulose membranes. Antibodies against ARID1A/BAF250a, IκBα, β-actin (Santa Cruz), phospho-IκBα, STAT3, phospho-STAT3 (Cell Signaling Technology) were used as the first antibodies. Anti-rabbit-800 or anti-mouse-800 secondary antibodies were used accordingly (Santa Cruz). Fluorescent intensity indicating protein expression levels were captured by Odyssey (LI-COR^®^ bioscience).

### Statistics

Statistical significance among groups was analyzed using either an unpaired two-sample Student’s *t*-test, one-way or two-way ANOVA. The *P* values for multiple comparisons were adjusted by Bonferroni correction method. We defined the statistical significance as a *P* value less than 0.05.

## Results

### Hepatocyte-specific *Arid* *1a* deficiency leads to mouse steatohepatitis and HCC

Like most human cancers, nonsense and frame shift mutations throughout *ARID1A* gene have frequently been found in HCC [18, 19, 22], indicating that these *ARID1A* mutations could inactivate *ARID1A* function in HCC. Base on this, we constructed conditional hepatocyte-specific *Arid1a* knockout (KO) (*Arid1a* ^*LKO*^) mice to explore its roles in HCC development by crossing mice carrying loxP-flanked *Arid1a* exon 8 alleles (*Arid1a* ^*f/f*^) with Alb-Cre transgenic mice, resulting in efficient Cre-mediated recombination in hepatocytes (S1A Fig). *Arid1a* ^*LKO*^ mice born viable and fertile at the expected Mendelian frequencies. Postnatal *Arid1a* ^*LKO*^ mice demonstrated efficient hepatic ablation of Arid1a/BAF250a (S1B Fig).

**Fig 1:**
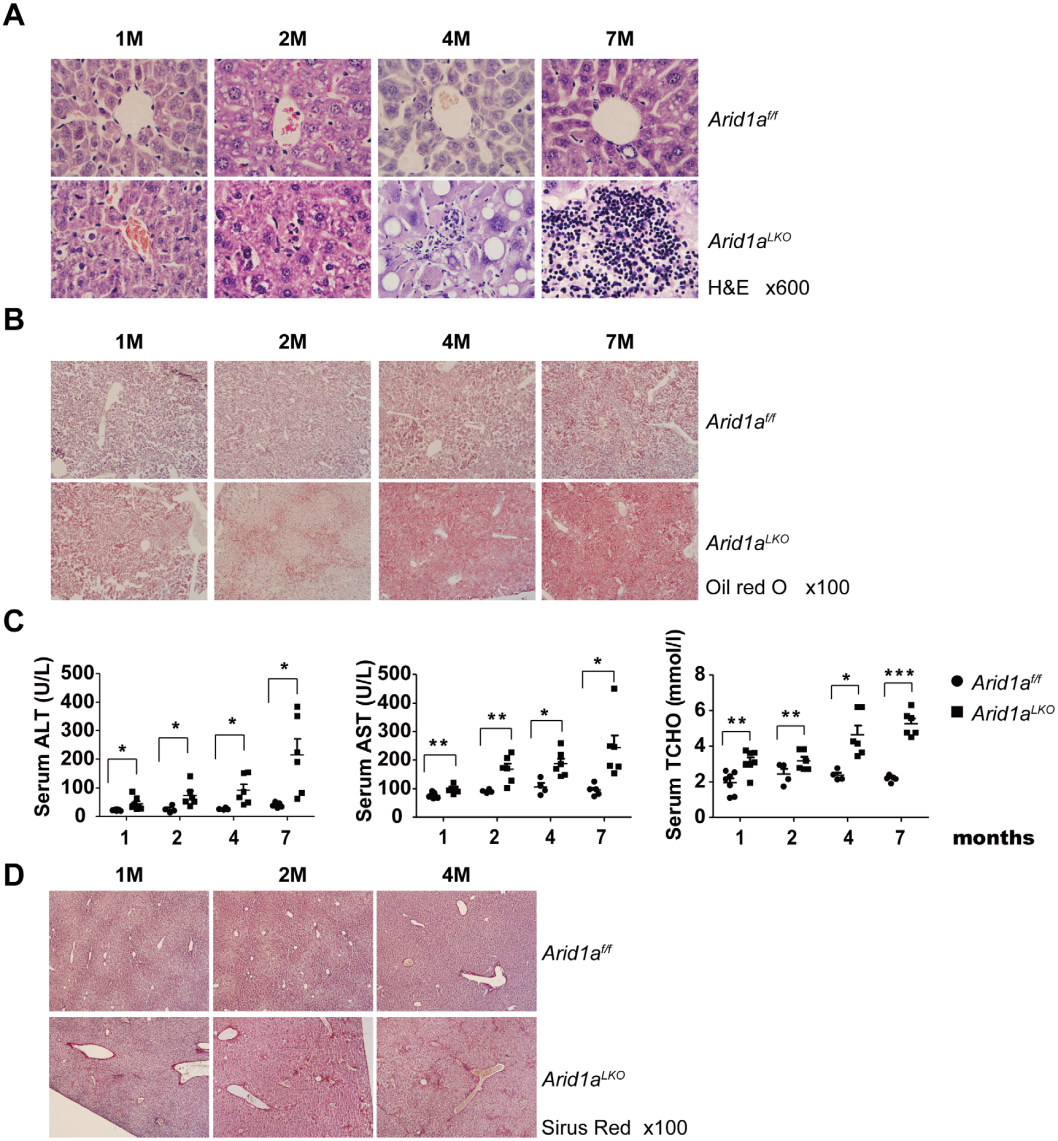
*Arid1a* deficiency promotes steatohepatitis development in *Arid1a* ^*LKO*^ mice. (A). Liver sections from 1, 2, 4 and 7 month (M) old *Arid1a* ^*LKO*^ mice and their littermates (*Arid1a* ^*f/f*^) as controls were stained using Hematoxylin and eosin (H&E). (B). Oil red O staining on liver sections from 1, 2, 4 and 7 month old *Arid1a* ^*LKO*^ mice and their *Arid1a* ^*f/f*^ littermates as controls. (C). Serum alanine aminotransferase (ALT), asparate aminotransferase (AST) and total cholesterol (TCHO) levels in 1, 2, 4 and 7 month old *Arid1a* ^*LKO*^ and *Arid1a* ^*f/f*^ mice. Each spot represents the measured value from each individual of two genotype groups, where each subgroup in the 4 different age groups contained a minimum of 4 individuals. The data are shown as the means ± SEM. Statistical significance among the experimental groups was assessed using an unpaired two-sample Student’s *t*-test. *P < 0.05; **P < 0.01; and ***P < 0.001. (D). Liver sections from 1, 2 and 4 month old *Arid1a* ^*LKO*^ mice and control littermates (*Arid1a* ^*f/f*^) were stained with Sirius red.

We examined the livers and serum indices of hepatic functions in *Arid1a* ^*LKO*^ mice at different ages. We found that infiltrating inflammatory cells and lipid accumulation, as revealed by oil red O staining, gradually increased in these mouse livers in an age-dependent manner (Fig 1A and 1B). Peripheral serum indices revealed that ALT, AST and TCHO were significantly increased in postnatal mice in an age-dependent manner (Fig 1C). Moreover, serum LDL-C, but not HDL-C, levels were also increased in *Arid1a* ^*LKO*^ mice (S1C Fig). These data indicate that steatohepatitis occurs in *Arid1a* ^*LKO*^ mice (Table 3). In addition, collagen deposition in the livers of 2 month old mice (Fig 1D), as indicated by Sirius red collagen staining, was also increased, suggesting that liver fibrogenesis occurred secondary to steatohepatitis.

**Table 3:** Summary of liver pathological examination in *Arid1a* deficient mice.

Age (month)	Steatosis		Ballooning		Lobular Inflammation		Portal Inflammation		NAI	
	*Arid1a* ^*f/f*^	*Arid1a* ^*LKO*^	*Arid1a* ^*f/f*^	*Arid1a* ^*LKO*^	*Arid1a* ^*f/f*^	*Arid1a* ^*LKO*^	*Arid1a* ^*f/f*^	*Arid1a* ^*LKO*^	*Arid1a* ^*f/f*^	*Arid1a* ^*LKO*^
1 (7,5)^#^	0.00	0.00	0.6±0.54	1.6±0.017*	1±0.71	1.6±0.09	0.2±0.45	1.6±0.001**	1.43±0.74	4.8±0.84***
4 (10,12)^#^	0.7±0.67	0.83±0.83	0.9±0.99	1.5±0.9**	1.3±1.15	2.5±0.9***	0.1±0.32	0.67±0.65**	2±1.53	6.3±1.3***
7 (10,7)^#^	0.6±0.51	1.14±1.06	0.5±0.52	1.42±1.13*	1.4±1.17	2.57±1.13	0.00	0.42±0.53*	2.7±1.3	5.57±2.07**
10 (17,20)^#^	0.65±0.71	1.55±1.09**	1.71±1.05	1.75±0.79	1.35±1.17	2.8±0.62***	0.35±0.078	1±0.46***	3.81±1.73	7.1±1.58***
>10 (13,15)^#^	0.54±0.66	1.67±1.18**	0.92±0.95	1.67±0.91*	0.92±0.95	2.53±0.92***	0.15±0.38	1.2±0.68***	2.53±1.06	7.06±2.05***

Pathological hepatic NAFLD (Non-alcoholic fatty liver disease) scores in each mouse group. The criteria for each score are described under Materials and Methods. NAI: NASH (nonalcoholic steatohepatitis) activity index, the sums of the four scores-steatosis, ballooning, lobular inflammation and portal inflammation. Results are showed as the mean ± s.d.

^#^ The numbers in parentheses indicate the mouse numbers of *Arid1a* ^*f/f*^ and *Arid1a* ^*LKO*^ groups, respectively.

* *P* < 0.05, *Arid1a* ^*LKO*^ *versus Arid1a* ^*f/f*^ mice.

** *P* < 0.01, *Arid1a* ^*LKO*^ *versus Arid1a* ^*f/f*^ mice.

*** *P* < 0.001, *Arid1a* ^*LKO*^ *versus Arid1a* ^*f/f*^ mice.

**Fig 2:**
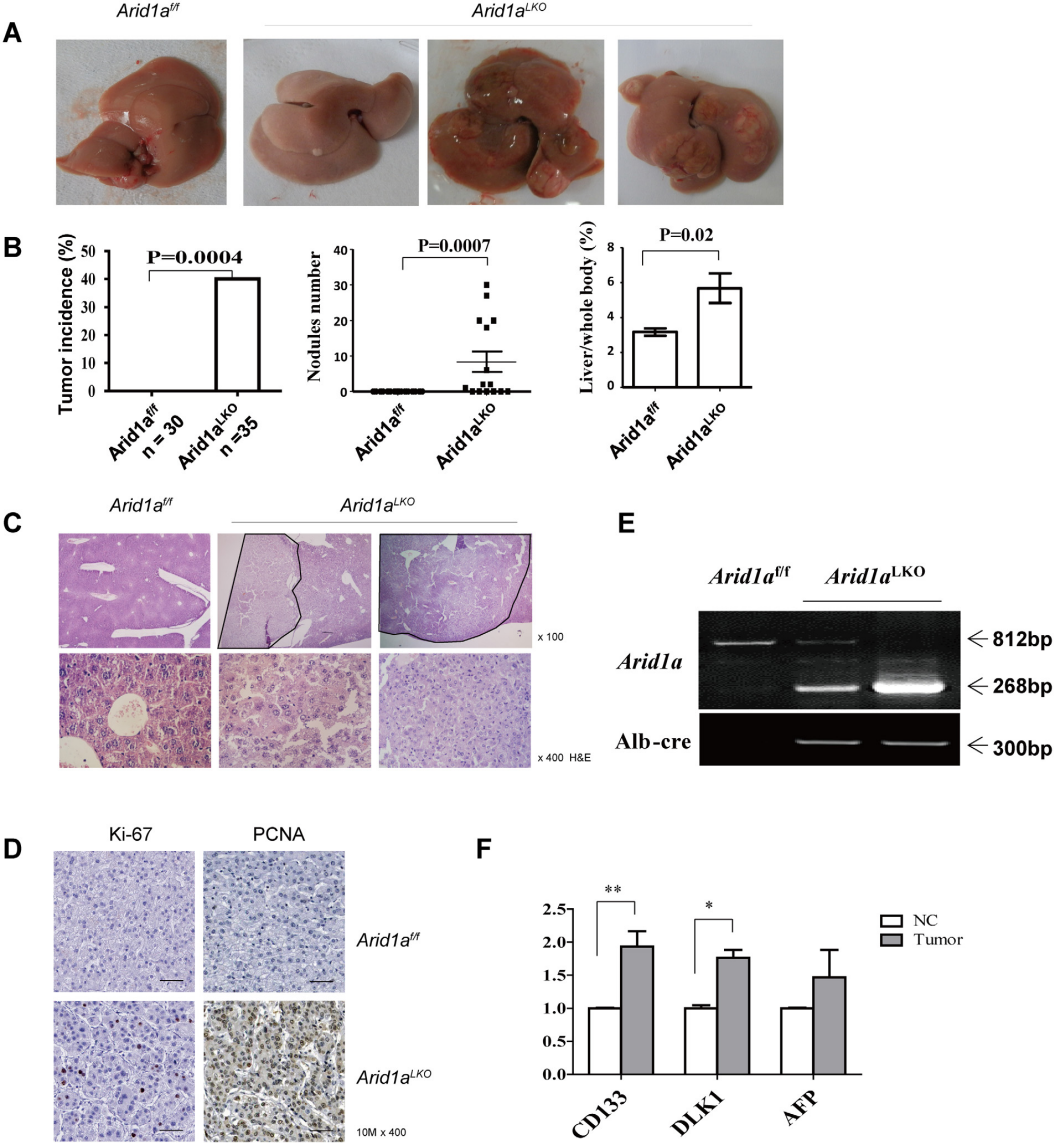
*Arid1a* deficiency promotes HCC development in *Arid1a* ^*LKO*^ mice. (A). Representative macroscopic livers from a 10 month old *Arid1a* ^*f/f*^ mouse (left), as well as 10, 14, and 17 month old *Arid1a* ^*LKO*^ male mice (right). Livers of *Arid1a* ^*LKO*^ mice show single or multiple tumor nodules. (B). Tumor incidence (left) was statistically analyzed by κ-test. Tumor nodule number (middle) and liver/body weight (right) were statistically analyzed using an unpaired two-sample Student’s *t*-test. The data are shown as the means ± SEM. *P* values are shown in the upper. (C). Representative microscopic histology of liver tumors in *Arid1a* ^*LKO*^ mice was examined under H&E staining by magnification of 100 (upper) and 400 times (lower), where the liver of an *Arid1a* ^*f/f*^ littermate was used as a control. (D). Immunohistochemical staining with antibodies raised against Ki-67 and PCNA was performed on liver tumor sections of 10 month old *Arid1a* ^*LKO*^ mice. A liver section from an *Arid1a* ^*f/f*^ littermate was used as a control. (E). DNA from HCC samples were harvested by Laser capture microdissection (LCM), and genotyping were performed with PCR. (F). Expression levels of *CD133*, *DLK1* and *AFP* were measured in tumors and the adjacent non-cancerous livers (NC) of *Arid1a* deficiency mice using quantitative RT-PCR. Each group contained a minimum of 3 individuals. The data are shown as the means ± SEM. Statistical analysis was performed using two-way ANOVA. *P < 0.05, **P < 0.01, and ***P < 0.001.

Interestingly, *Arid1a* ^*LKO*^ hepatocytes displayed characteristic features of large-cell dysplasia with strong anisokaryosis (S1D Fig), which was associated with an increased risk of HCC development [36, 37]. About forty percent of these 10–18 month old male *Arid1a* ^*LKO*^ mice developed macroscopically visible liver tumors (Fig 2A and 2B and Table 3), whereas no liver tumors were found in *Arid1a* ^*f/f*^ littermate controls. Histological analyses revealed that all of these liver tumors were HCCs (Fig 2C). These HCCs were characterized by expansive growth, increased cellularity and neutrophil infiltration and the absence of portal tracts, as compared to non-tumorous areas. Immunohistochemical (IHC) staining revealed that these HCCs exhibited significant cell proliferation, as indicated by elevated Ki-67 and PCNA expression (Fig 2D). Interestingly, PCNA positivity was much higher than Ki67 positivity in the pathological examination. Although both PCNA and Ki-67 are biomarkers for cell proliferation in tumors, PCNA is an accessory protein for DNA polymerase-alpha required for DNA synthesis, and has a key role in cell cycle initiation, while the expression of Ki-67 reflects the number of proliferating cells in a tissue. Non-dividing or “resting” cells in the G0 phase are Ki-67 antigen negative. Thus, the finding that PCNA positivity was much higher than Ki67 positivity in the *Arid1a* ^*LKO*^ mice reflects that the more DNA replication and mitotic activity in G0/S phases, not cell division, occurs in HCC cells in the absence of *Arid1a*. Genotypes of these HCC samples, including tumor cells harvested by LCM, showed that these tumors were mainly composed of *Arid1a*-deficient cells (Fig 2E). Some known molecular markers CD133, AFP and DLK1 were upregulated in these tumors (Fig 2F).

### Arid1a deficiency enhances diethylnitrosamine (DEN)-induced hepatocarcinogenesis

**Fig 3:**
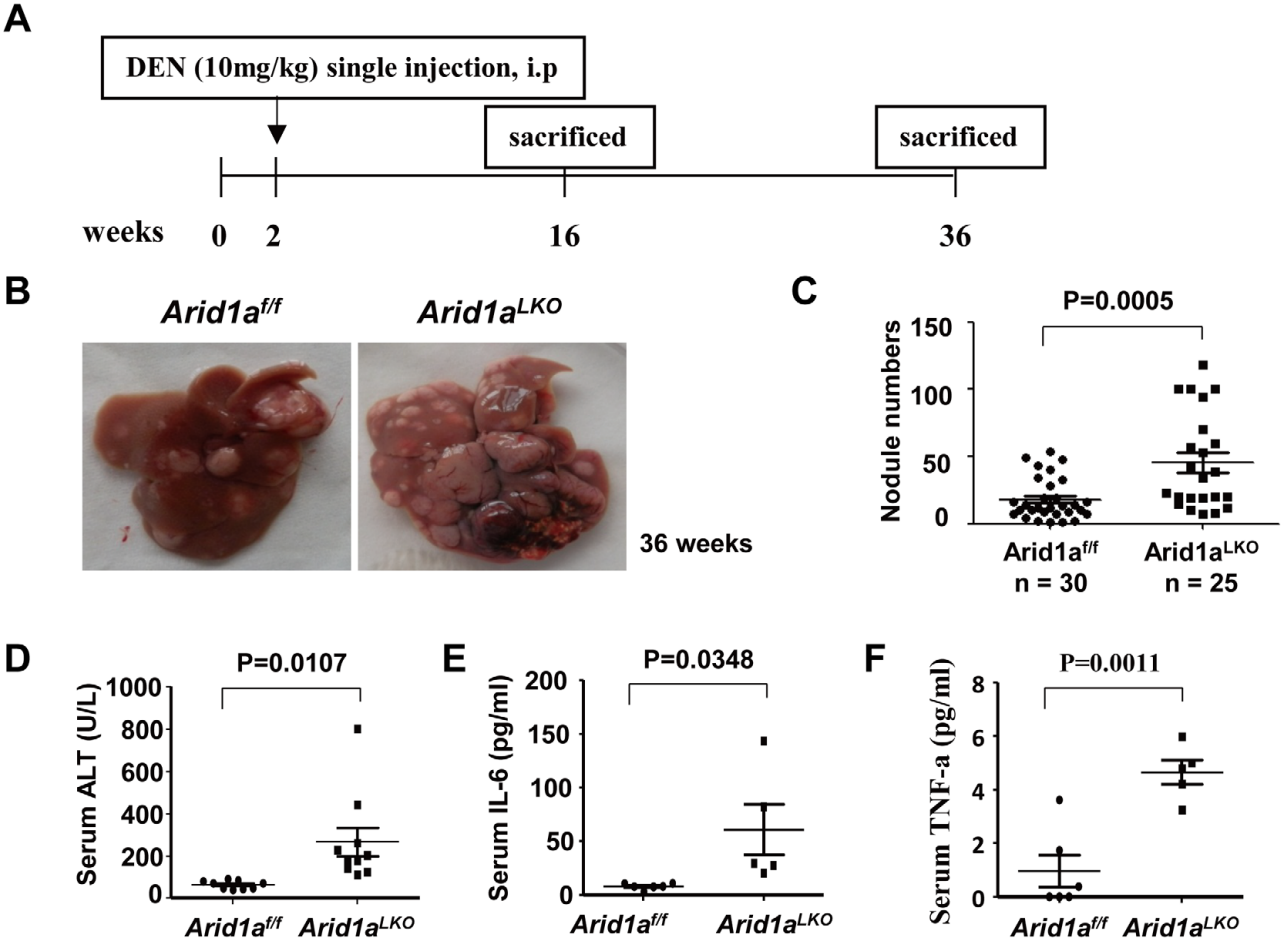
Increased HCC tumorigenesis in DEN-treated *Arid1a* ^*LKO*^ mice (A). A brief scheme illustrating DEN-induced HCC experiments in *Arid1a* ^*LKO*^ mice and *Arid1a* ^*f/f*^ littermates. (B). Representative macroscopic livers from 9 month old male *Arid1a* ^*f/f*^ (left) and *Arid1a* ^*LKO*^ mice (right). Multiple larger tumor nodules on the surface of the livers of *Arid1a* ^*LKO*^ mice are shown. (C). Tumor nodules were counted and statistically analyzed using an unpaired two-sample Student’s *t*-test. The data are shown as the means ± SEM. The *P* value is shown above compared groups. (D-F). Serum ALT (D), IL-6 (E) and TNF-α (F) levels in 4 month old *Arid1a* ^*LKO*^ and *Arid1a* ^*f/f*^ mice. Each spot represents the measured value from each individual of two genotype groups, where each group contains a minimum of 5 individuals. The data are shown as the means ± SEM. Statistical analysis was performed using an unpaired two-sample Student’s *t*-test. *P* values are shown above compared groups.

In order to further ascertain the role of *Arid1a* in liver tumorigenesis, we analyzed the susceptibility of *Arid1a* ^*LKO*^ mice to the carcinogen DEN. DEN is used to generate a multistage hepatocarcinogenesis murine model, which might partly mimic human HCC tumorigenesis [38, 39]. Significantly, a single intraperitoneal injection of DEN increased tumor incidence and the number of macroscopic tumor nodules on the liver surface of *Arid1a* ^*LKO*^ mice at the ninth months after DEN administration as compared to *Arid1a* ^*f/f*^ littermates (Fig 3A–3C), revealing that *Arid1a* deficiency enhances DEN-induced hepatocarcinogenesis. Levels of serum ALT in *Arid1a* ^*LKO*^ mice following DEN treatment were significantly higher than those in *Arid1a* ^*f/f*^ littermates (Fig 3D), indicating that *Arid1a-deficient* mice were sensitive to DEN-induced hepatic damage and inflammation. Additionally, serum IL-6 and TNF-α were also significantly elevated in 4 month old *Arid1a* ^*LKO*^ mice (Fig 3E and 3F). These data suggest that *Arid1a* deficiency may enhance DEN-induced hepatocarcinogenesis by enhancing hepatic damage and liver inflammation, which are accompanied by excessive proinflammatory cytokine production.

### Increased proinflammatory cytokine release and innate immune cell infiltration in the livers of Arid1a-deficient mice

**Fig 4:**
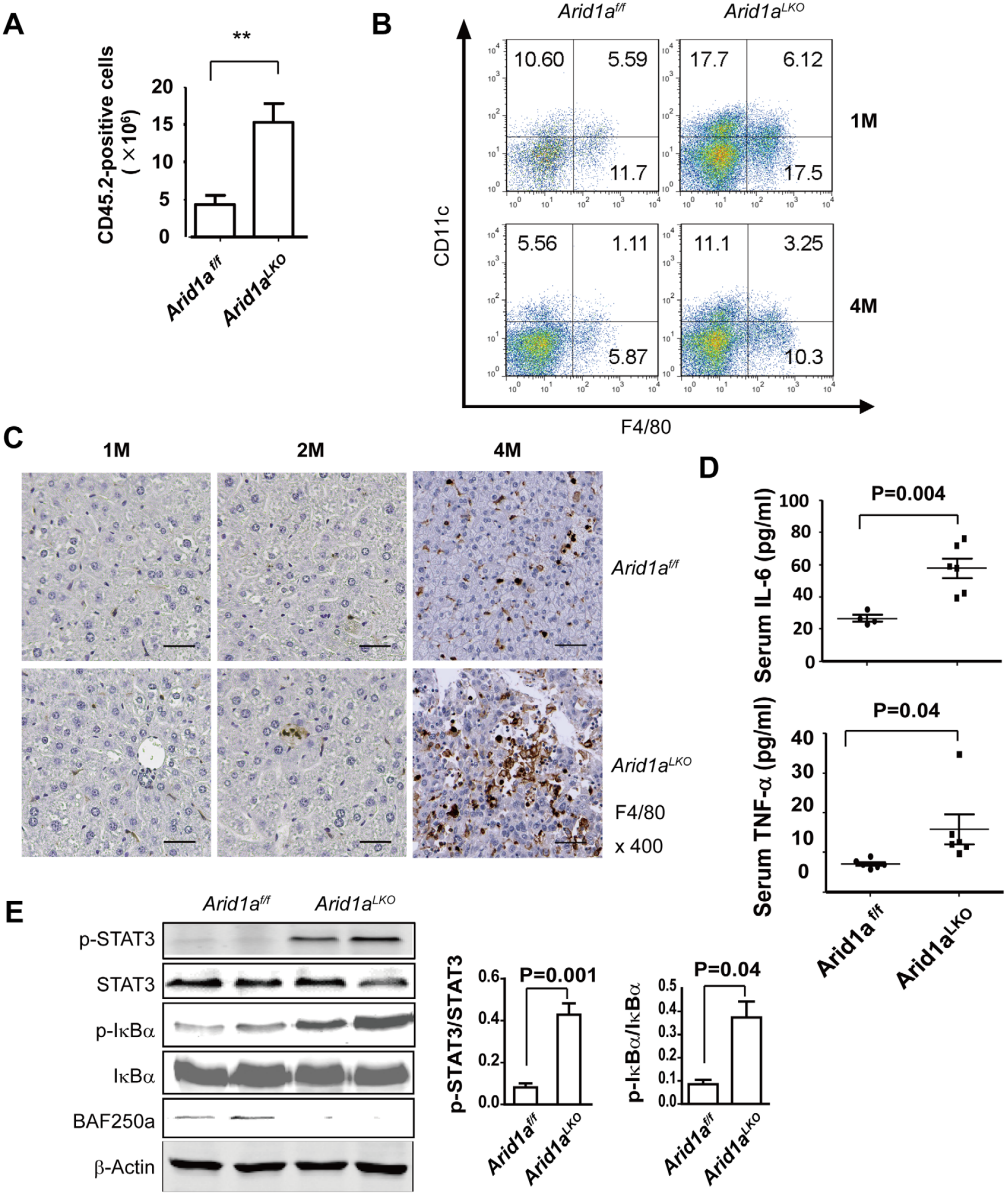
Innate immune cell infiltration in livers of *Arid1a* ^*LKO*^ mice. (A). Flow cytometric (FACS) analysis of liver nonparenchymal cells (NPCs) from 1 month old *Arid1a* ^*LKO*^ and *Arid1a* ^*f/f*^ mice was performed using an anti-CD45.2 antibody to count the number of infiltrated CD45-positive leukocytes in the livers. The data are shown as the means ± SEM. Statistical analysis was performed using an unpaired two-sample Student’s *t*-test. **P < 0.01. (B). FACS analysis of liver NPCs from 1 and 4 month old *Arid1a* ^*LKO*^ and *Arid1a* ^*f/f*^ mice was used to assess macrophage and neutrophil numbers with anti-F4/80 and CD11c antibodies, respectively. The numbers represent the percentages of macrophages or neutrophil leukocytes in liver NPCs. (C). Representative Immunohistochemical analysis using an anti-F4/80 antibody on liver sections from 1, 2 and 4 month old *Arid1a* ^*LKO*^ and *Arid1a* ^*f/f*^ mice. (D). Serum IL-6 and TNF-α levels in 1 month old *Arid1a* ^*LKO*^ mice and their *Arid1a* ^*f/f*^ littermates. Each spot represents the measured value from each individual of two genotype groups, in which each group contained 5–7 individuals. The data are shown as the means ± SEM. Statistical analysis was performed using an unpaired two-sample Student’s *t*-test. *P* values are shown above compared groups. (E). Western blot analysis of protein extracts from the livers of 1 month old *Arid1a* ^*LKO*^ and *Arid1a* ^*f/f*^ mice was performed using anti-STAT3, phosphorylated-STAT3, IκBα and phosphorylated-IκBα antibodies. Relative phosphorylation levels were shown on the right. The phosphorylation levels of the proteins were evaluated based on intensity of their bands. Each group has 6 mice, and the represented data was showed. BAF250a was used to evaluate the samples and β-actin was used as an internal control. The data are shown as the means ± SEM. Statistical analysis was performed using an unpaired two-sample Student’s *t*-test. *P* values are shown above compared groups.

Chronic liver inflammation is a common trigger of liver diseases, including liver fibrogenesis and HCC development [40, 41]. Innate immune cells may initiate and maintain hepatic inflammation responses via proinflammatory cytokine production [42]. In the present study, we found that 1–7 month old *Arid1a* ^*LKO*^ mice displayed liver inflammation that was characterized by elevated serum ALT levels (Fig 1C) and immune cell infiltration into the liver parenchyma (Fig 1A). These findings were supported by flow cytometric analyses indicating that CD45-positive leukocytes were significantly increased in these mice livers (Fig 4A). Flow cytometry was further employed to distinguish infiltrated inflammatory cells. As shown in Fig 4B and S2A Fig, F4/80^+^ macrophages and CD11c^+^ neutrophil cells, but not CD19- and CD3-positive cells, were increased in the livers of 1 and 4 month old *Arid1a* ^*LKO*^ mice as compared to their *Arid1a* ^*f/f*^ littermates. IHC staining also revealed that the levels of F4/80^+^ macrophages gradually increased in the livers of 1–4 month old *Arid1a* ^*LKO*^ mice (Fig 4C). These data indicated that the livers of *Arid1a* ^*LKO*^ mice were infiltrated by innate immune cells.

We next assessed serum levels of the proinflammatory cytokines IL-6 and TNF-α, which are considered to be the most important cytokines that promote HCC tumorigenesis [43, 44]. Significantly, these two proinflammatory cytokines were markedly elevated in *Arid1a* ^*LKO*^ mice (Fig 4D). We further measured the transcripts of some known proinflammatory cytokines and chemokines in the livers of the mice using real-time RT-PCR. In addition to *IL-6* and *TNF-α*, mRNA levels of *IFN-γ*, *Ccl1*, *Ccl9*, *Ccl12*, and *Cxcl11* were also significantly elevated in *Arid1a* ^*LKO*^ mice as compared to their littermates (S2B Fig). These data suggested that proinflammatory cytokines and chemokines were maladjusted in *Arid1a* ^*LKO*^ mice.

As previously reported, increased proinflammatory cytokine and/or chemokine production may activate the STAT3 and NF-κB pathways, which are known to be closely associated with HCC development [45]. In the present study, we further examined the intracellular STAT3 and NF-κB pathways by detecting phosphorylated STAT3 and IκBα. As expected, phosphorylated STAT3 and IκBα were obviously elevated in the livers of *Arid1a* ^*LKO*^ mice (Fig 4E), suggesting that the STAT3 and NF-κB pathways could be activated by the release of proinflammatory cytokines and/or chemokines, which may further lead to HCC development in the livers of *Arid1a* ^*LKO*^ mice.

**Fig 5:**
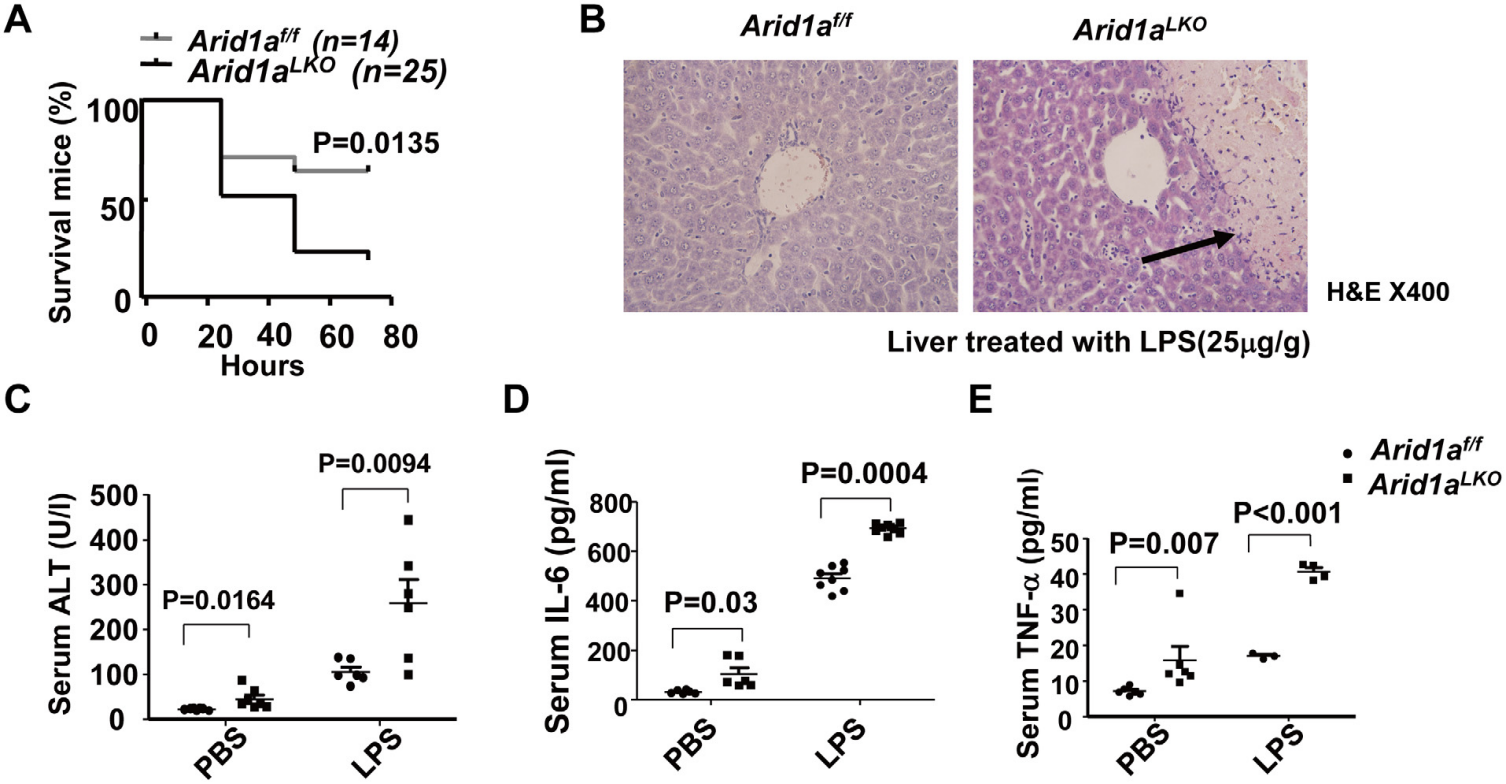
*Arid1a* deficiency enhances LPS induced hepatitis. (A). LPS lethality experiments in *Arid1a* ^*LKO*^ and *Arid1a* ^*f/f*^ mice. Survival curves were statistically analyzed using two-way ANOVA. (B). H&E staining on livers from LPS-treated *Arid1a* ^*LKO*^ and *Arid1a* ^*f/f*^ mice. (C-E). Serum ALT (C), IL-6 (D) and TNF-α (E) levels were measured in *Arid1a* ^*LKO*^ and *Arid1a* ^*f/f*^ mice following LPS administration, where PBS was used as a negative control. Each spot represents the measured value from each individual of two genotype groups, in which each group contained 3–8 individuals. The data are shown as the means ± SEM. Statistical analysis was performed using an unpaired two-sample Student’s *t*-test. *P* values are shown above compared groups.

Lipopolysaccharide (LPS) has been shown to activate macrophages and neutrophils to release proinflammatory factors by binding to TLR4, and recent studies have revealed that LPS from Gram-negative bacteria in the gut flora may enhance liver inflammation, hepatic damage and HCC promotion [46, 47]. To further assess whether LPS can enhance hepatic damage and liver inflammation, *Arid1a* ^*LKO*^ mice were treated with a single LPS dose intraperitoneally. Interestingly, 48 hours after LPS administration, the death rate of these mice was significantly higher than that of their *Arid1a* ^*f/f*^ littermates (Fig 5A). We also examined the livers of *Arid1a* ^*LKO*^ mice after LPS administration, which exhibited massive necrosis at 24 hours (Fig 5B). In parallel, serum ALT levels in *Arid1a* ^*LKO*^ mice were significantly higher than those of their *Arid1a* ^*f/f*^ littermates after lower dose LPS administration (Fig 5C). Interestingly, serum IL-6 and TNF-α were significantly elevated in *Arid1a* ^*LKO*^ mice (Fig 5D and 5E). These data suggested that LPS from the gut flora could enhance hepatic damage and liver inflammation in *Arid1a* ^*LKO*^ mice, possibly by triggering macrophage and neutrophil infiltration in the liver, thereby promoting HCC development in *Arid1a-deficient* mice [46].

## Discussion

Human HCC has been well known to be closely associated with multiple risk factors, such as persistent hepatitis infection, chronic alcohol consumption and aflatoxin B1 exposure [48, 49]. Additionally, metabolic disorders, such as diabetes and obesity, are also considered as risk factors for liver cancer [50, 51]. Regardless of the etiology, the neoplastic lesions usually originate on a bed of chronic inflammation that sequentially progresses from fibrosis to cirrhosis and finally culminates in HCC [49, 52]. It is general recognized that chronic inflammation is closely associated with chronic liver injure, including apoptosis and/or necrosis.

As with any other neoplasia, HCC development also involves a series of genetic alterations, particularly somatic mutations. In addition to TERT [53], TP53 and β-catenin mutations, some genes encoding components of SWI/SNF complexes, such as ARID1A and ARID2, were also found to be frequently mutated in HCC [18, 19, 22]. However, the role and mechanisms underlying their loss of function mutations in HCC development are completely unclear. The clarification of a causal relationship between somatic mutations of SWI/SNF chromatin remodeling molecules and HCC development, including chronic liver damage and inflammation that remodel the pro-carcinogenic microenvironment, will be helpful in understanding the tumorigenic process.

The SWI/SNF chromatin remodeling complex, which have helicase and ATPase activities, regulates gene transcription by altering the chromatin structure [54]. Recently, certain genes encoding components of SWI/SNF complexes, in particular *ARID1A*, have been reported to be frequently mutated in a wide variety of human cancers [55]. Based on genetically engineered mouse models, compelling evidence indicates that deficiencies in certain components of SWI/SNF complexes may contribute to tumor development. BRG1 (a core member of SWI/SNF complexes) haploinsufficient mice displayed a mildly tumor prone phenotype, with 10% of mice developing glandular tumors [55]. SNF (another core subunit of SWI/SNF complexes) deficient mice also developed lymphoma and pancytopenia [56]. In ovarian cancer, *ARID1A* deficiency alone is not sufficient for promoting tumorigenesis, it requires PIK3CA co-activation [24, 25]. In the present study, we demonstrated that hepatocyte-specific *Arid1a* deficiency results in HCC development in a murine model. Significantly, as with human HCC, animals in the *Arid1a* deficiency-induced murine HCC model undergo chronic liver damage and inflammation, steatohepatitis, hepatocyte dysplasia, and ultimately develop HCC, implying that human ARID1A loss of function mutations or decreases may be a critical event that triggers a cascade culminating in HCC development. These data also suggest that chronic liver damage and inflammation induced by inactivated and dysregulated ARID1A may be an early requirement for HCC development.

In the *Arid1a* deficiency-driven HCC model, we found that chronic inflammation accumulates in hepatic lobules, which was characterized by the increased macrophages infiltration, the elevated cytokines such as IL-6 and TNF-α and chemokines, and the activated JAK-STAT3 and NF-κB signaling pathways. The liver inflammation with excess proinflammation cytokines shape cancer-promoting microenvironment. It should be pointed out that IL-6 has been proven to be closely associated with the development of hepatic steatosis and inflammation [57]. Previous studies have demonstrated that IL-6 produced by Kupffer cells or macrophages plays a crucial role in HCC development [41, 58]. The hepatic progenitor cells were expanded through the acquired autocrine IL-6 signaling that stimulates malignant transformation and progression of HCC [59]. The underlying mechanism involved in the *Arid1a* deficiency-driven HCC could be associated with the activated IL-6 signaling. A recent report also revealed that both *Arid1a* and *PIK3CA* mutations may cooperatively promote tumour development through the sustained IL-6 overproduction in ovary cancer, indicated by a mouse model [24, 25], where *Arid1a* protects against the inflammation-driven tumorigenesis, which is similar to our found in the *Arid1a* deficiency-driven HCC. Recently, IL-6 and some components involved in IL-6/STAT3 pathway are considered as the therapeutic targets, because the inhibition of IL-6/STAT3 pathway may attenuate the HCC cell survival upon IL-6 production [60].

Apart from the activated IL-6/STAT3 pathway, *Arid1a* deficiency also may disrupt the structure and functions of SWI/SNF chromatin remodeling complex, leading to genomic instability, which could contribute to HCC tumorigenesis. However, the detail underlying mechanism involved in genomic instability and cancer-promoting microenvironment in the *Arid1a* deficiency-driven HCC model should be further investigated.

In conclusion, *Arid1a* deficiency leads to the stimulation of innate immune cells, including monocytes, Kupffer cells and neutrophils, to produce proinflammatory cytokines, such as IL-6 and TNF-α, which promote steatohepatitis and HCC development.

## Supporting Information

S1 Fig *Arid1a* deficiency promotes steatohepatitis. (A). Genotypes of *Arid1a* ^*LKO*^ and *Arid1a* ^*f/f*^ mice were identified in livers by PCR. (B). Arid1a/BAF250 protein expression in livers of *Arid1a* ^*LKO*^ and *Arid1a* ^*f/f*^ mice was evaluated by Western blotting assay. (C). LDL-C and HDL-C levels in 1-month-old *Arid1a* ^*LKO*^ and *Arid1a* ^*f/f*^ mice. The data are shown as the means ± SEM. Statistical significance among the experimental groups was assessed using an unpaired two-sample Student’s *t*-test. **P < 0.01. (D). Liver sections from 1, 2, 4 and 7 month old *Arid1a* ^*LKO*^ mice and their *Arid1a* ^*f/f*^ littermates as controls were stained with H&E.
(TIF)

S2 Fig Enhanced inflammatory response in *Arid1a* ^*LKO*^ mice. (A). FACS analysis of NPCs from 1-month-old and 4-months-old *Arid1a* ^*LKO*^ and *Arid1a* ^*f/f*^ mouse livers was performed with anti-CD3 and CD19 fluorescent conjugated antibodies. (B). The mRNA expression levels of some cytokines and chemokines were detected in livers from 4-weeks-old *Arid1a* ^*LKO*^ and *Arid1a* ^*f/f*^ mice by quantitative real-time RT-PCR. The mRNA expression levels from *Arid1a* ^*f/f*^ mice were normalized as control. Results are shown as mean, error bars indicate standard error of the mean (SEM). *P < 0.05; **P < 0.01(n = 3 each genotype).
(TIF)
