# Supplementary figures and images for: Hepatocyte-Specific Arid1a Deficiency Initiates Mouse Steatohepatitis and Hepatocellular Carcinoma
Source: PLoS One. 2015 Nov 16;10(11):e0143042. doi: 10.1371/journal.pone.0143042 (PMC4646347; doi:10.1371/journal.pone.0143042)

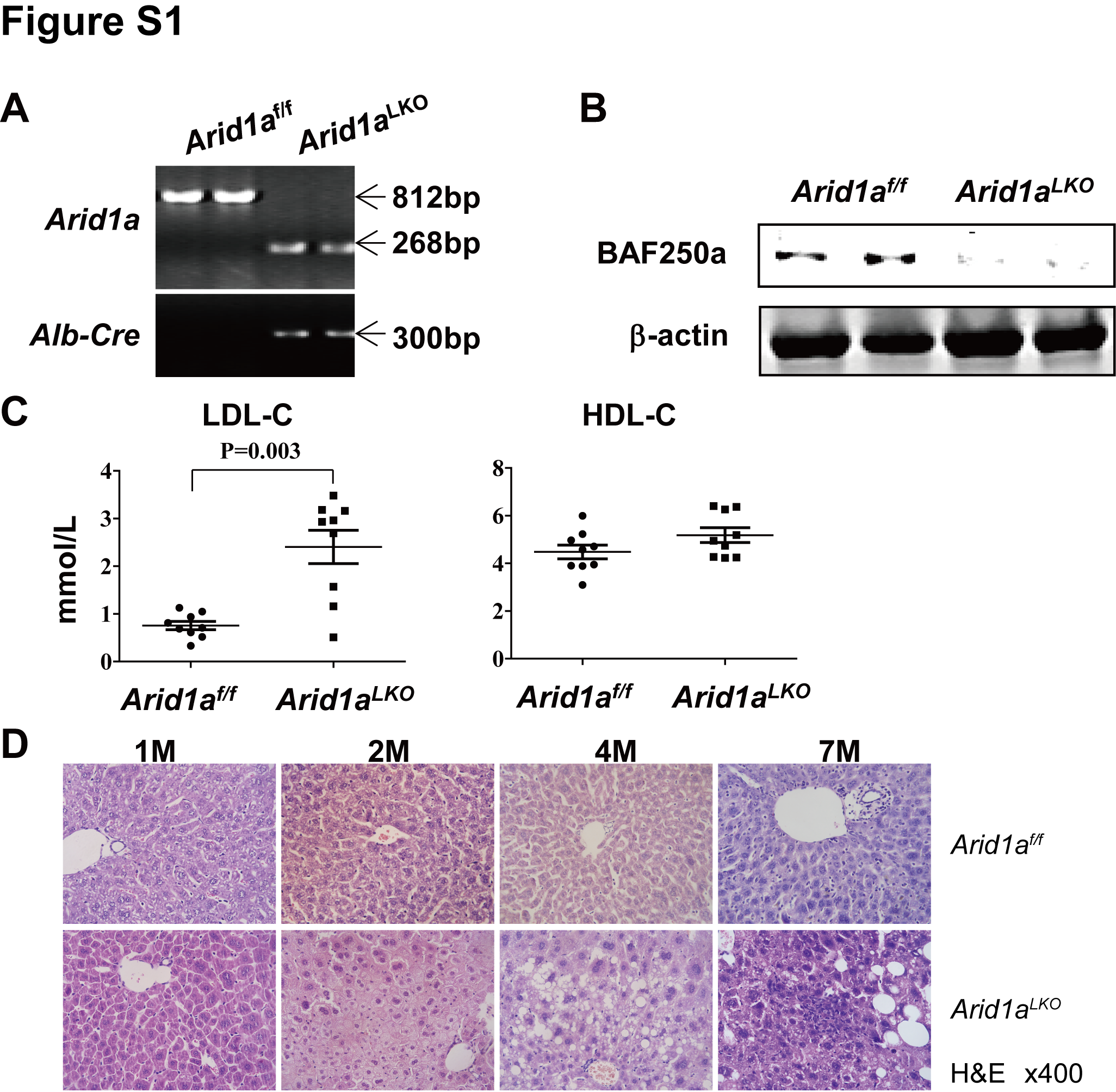

Supplement: S1 Fig — (A). Genotypes of Arid1a LKO and Arid1a f/f mice were identified in livers by PCR. (B). Arid1a/BAF250 protein expression in livers of Arid1a LKO and Arid1a f/f mice was evaluated by Western blotting assay. (C). LDL-C and HDL-C levels in 1-month-old Arid1a LKO and Arid1a f/f mice. The data are shown as the means ± SEM. Statistical significance among the experimental groups was assessed using an unpaired two-sample Student’s t-test. **P < 0.01. (D). Liver sections from 1, 2, 4 and 7 month old Arid1a LKO mice and their Arid1a f/f littermates as controls were stained with H&E. (TIF) [file pone.0143042.s001.tif]

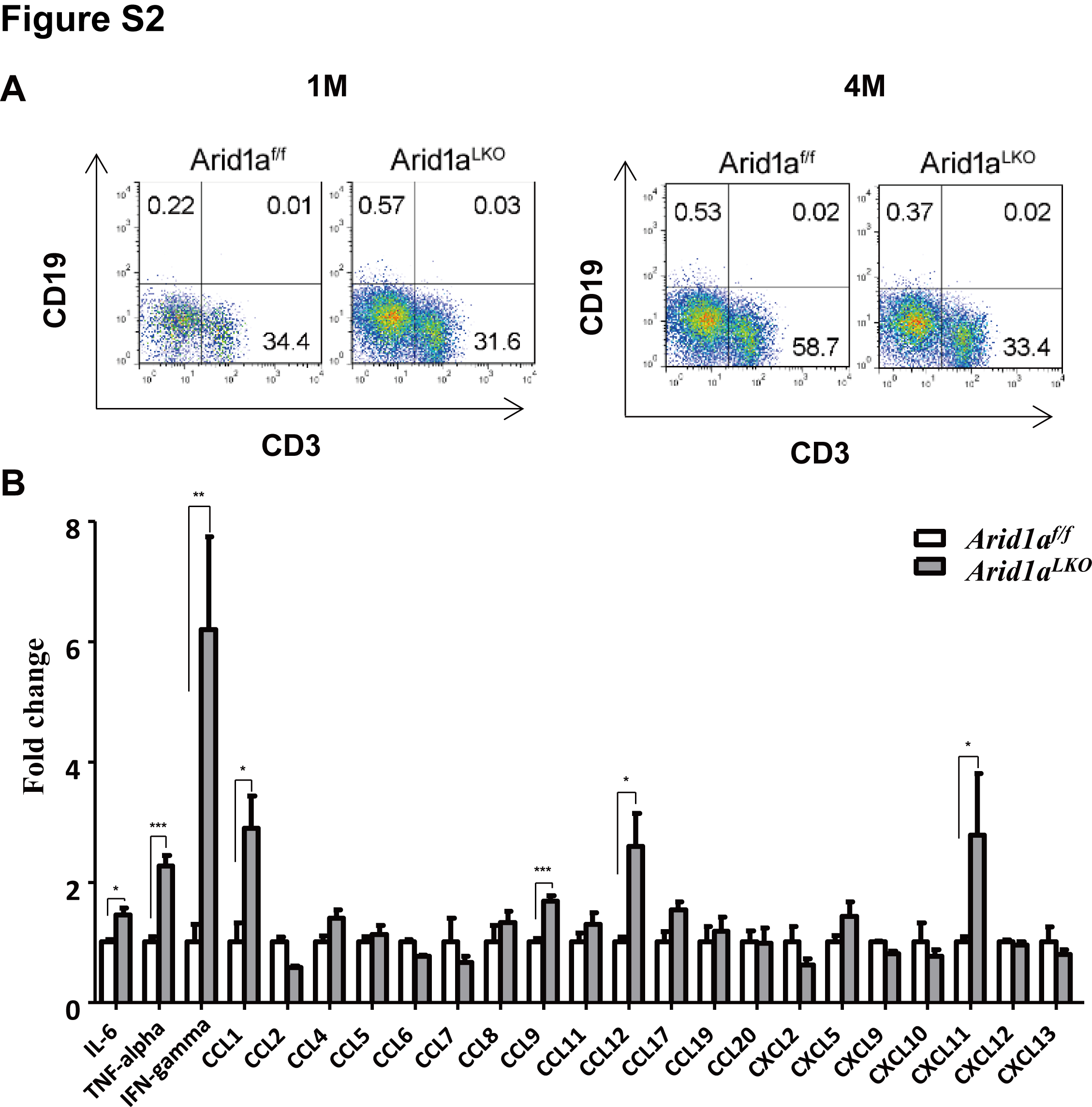

Supplement: S2 Fig — (A). FACS analysis of NPCs from 1-month-old and 4-months-old Arid1a LKO and Arid1a f/f mouse livers was performed with anti-CD3 and CD19 fluorescent conjugated antibodies. (B). The mRNA expression levels of some cytokines and chemokines were detected in livers from 4-weeks-old Arid1a LKO and Arid1a f/f mice by quantitative real-time RT-PCR. The mRNA expression levels from Arid1a f/f mice were normalized as control. Results are shown as mean, error bars indicate standard error of the mean (SEM). *P < 0.05; **P < 0.01(n = 3 each genotype). (TIF) [file pone.0143042.s002.tif]
